# CD3, CD8, IFN-γ, tumor and stroma inflammatory cells as prognostic indicators for surgically resected SCLC: evidences from a 10-year retrospective study and immunohistochemical analysis

**DOI:** 10.1007/s10238-024-01329-9

**Published:** 2024-05-15

**Authors:** Meng Fu, Chunmei Feng, Jialiang Wang, Chang Guo, Yongguang Wang, Rong Gao, Jiexiao Wang, Qizhi Zhu, Xiaopeng Zhang, Jian Qi, Yani Zhang, Yuting Bian, Zhipeng Wang, Yuan Fang, Lejie Cao, Bo Hong, Hongzhi Wang

**Affiliations:** 1 Hefei Cancer Hospital of CAS, Institute of Health and Medical Technology, Hefei Institutes of Physical Science, Chinese Academy of Sciences (CAS), Hefei, 230031 Anhui China; 2 Science Island Branch, Graduate School of University of Science and Technology of China, Hefei, 230026 Anhui China; 3 Department of Pulmonary and Critical Care Medicine, The First Affiliated Hospital of USTC, Division of Life Science and Medicine, University of Science and Technology of China (USTC), Hefei, 230001 Anhui China; 4 School of Basic Medicine, Anhui Medical University, Hefei, 230032 Anhui China; 5 Department of Pulmonary and Critical Care Medicine, Shanghai East Hospital, Tongji University School of Medicine, Shanghai, 200120 China

**Keywords:** Small cell lung cancer, Surgery, Tumor inflammatory cell, Stroma inflammatory cell, IFN-γ, CD3, CD8, Prognosis, Lymphatic metastasis

## Abstract

Current clinical guidelines limit surgical intervention to patients with cT1-2N0M0 small cell lung cancer (SCLC). Our objective was to reassess the role of surgery in SCLC management, and explore novel prognostic indicators for surgically resected SCLC. We reviewed all patients diagnosed with SCLC from January 2011 to April 2021 in our institution. Survival analysis was conducted using the Kaplan–Meier method, and independent prognostic factors were assessed through the Cox proportional hazard model. In addition, immunohistochemistry (IHC) staining was performed to evaluate the predictive value of selected indicators in the prognosis of surgically resected SCLC patients. In the study, 177 SCLC patients undergoing surgical resection were ultimately included. Both univariate and multivariate Cox analysis revealed that incomplete postoperative adjuvant therapy emerged as an independent risk factor for adverse prognosis (*p* < 0.001, HR 2.96). Survival analysis revealed significantly superior survival among pN0-1 patients compared to pN2 patients (*p* < 0.0001). No significant difference in postoperative survival was observed between pN1 and pN0 patients (*p* = 0.062). Patients with postoperative stable disease (SD) exhibited lower levels of tumor inflammatory cells (TIC) (*p* = 0.0047) and IFN-γ expression in both area and intensity (*p* < 0.0001 and 0.0091, respectively) compared to those with postoperative progressive disease (PD). Conversely, patients with postoperative SD showed elevated levels of stromal inflammatory cells (SIC) (*p* = 0.0453) and increased counts of CD3^+^ and CD8^+^ cells (*p* = 0.0262 and 0.0330, respectively). Survival analysis indicated that high levels of SIC, along with low levels of IFN-γ^+^ cell area within tumor tissue, may correlate positively with improved prognosis in surgically resected SCLC (*p* = 0.017 and 0.012, respectively). In conclusion, the present study revealed that the patients with pT1-2N1M0 staging were a potential subgroup of SCLC patients who may benefit from surgery. Complete postoperative adjuvant therapy remains an independent factor promoting a better prognosis for SCLC patients undergoing surgical resection. Moreover, CD3, CD8, IFN-γ, TIC, and SIC may serve as potential indicators for predicting the prognosis of surgically resected SCLC.

**Supplementary Information:**

The online version contains supplementary material available at 10.1007/s10238-024-01329-9.

## Introduction

According to Global Cancer Statistics 2020 (GLOBOCAN Estimates) by the International Agency for Research on Cancer (IARC), lung cancer was the most common malignant tumor worldwide with the highest mortality rate (18%), and the incidence rate (11.4%) was second only to female breast cancer [1]. Small cell lung cancer (SCLC) is a high-grade aggressive neuroendocrine malignancy, accounting for about 10–15% of all types of lung cancer [2, 3]. Given the characteristics of high metastatic potential and an easy relapse tendency of SCLC, it has long been considered to be associated with invasive clinical features and generally poor prognosis, even after surgery [4, 5]. Considering the discouraging overall prognosis and unmet treatment need for SCLC, increasing the surgical resection rate and identifying novel prognostic indicators are essential measures to improve patients survival.

Currently, clinical guidelines recommend surgical intervention exclusively for stage I-IIA (cT1-2N0M0) SCLC. However, less than 5% of SCLC cases were initially diagnosed at this stage [2, 6]. Numerous studies have provided evidences that the optimal approach for SCLC patients, diagnosed with the mentioned clinical staging, involves surgical resection combined with postoperative adjuvant chemotherapy and concurrent radiotherapy, resulting in maximum benefits [7–11]. Noteworthy, in light of recent non-randomized data, some SCLC patients with IIB-III staging may also benefit from surgical resection. These suggests that the role of surgery in the treatment of SCLC may have been underestimated and needs to be carefully reconsidered in conjunction with clinical practice and randomized controlled trials (RCTs) [7, 9, 12].

The implementation of high-resolution computed tomography (CT) screening for lung cancer is expected to increase the number of potentially operable cases of SCLC [10]. Furthermore, the clinical utilization of surgical intervention for SCLC is increasing, propelled by the establishment of multidisciplinary teams (MDTs) across thoracic surgery, respiratory medicine, oncology, and radiation therapy departments in numerous hospitals. In this study, we conducted a retrospective analysis encompassing 177 surgically treated SCLC patients at the First Affiliated Hospital of University of Science and Technology of China (USTC) from 2010 to 2021. Immunohistochemistry (IHC) staining were conducted on surgical samples from 18 patients to assess the expression levels of CD3, CD8, CD31, interferon-gamma (IFN-γ), tumor necrosis factor-alpha (TNF-α), and programmed death ligand-1 (PD-L1). Our objective was to reassess the role of surgery in SCLC management, explore whether SCLC patients with positive lymph nodes benefit from surgery, and identify novel prognostic indicators for surgically resected SCLC.

## Methods

### Research objects and study design

**Fig. 1:**
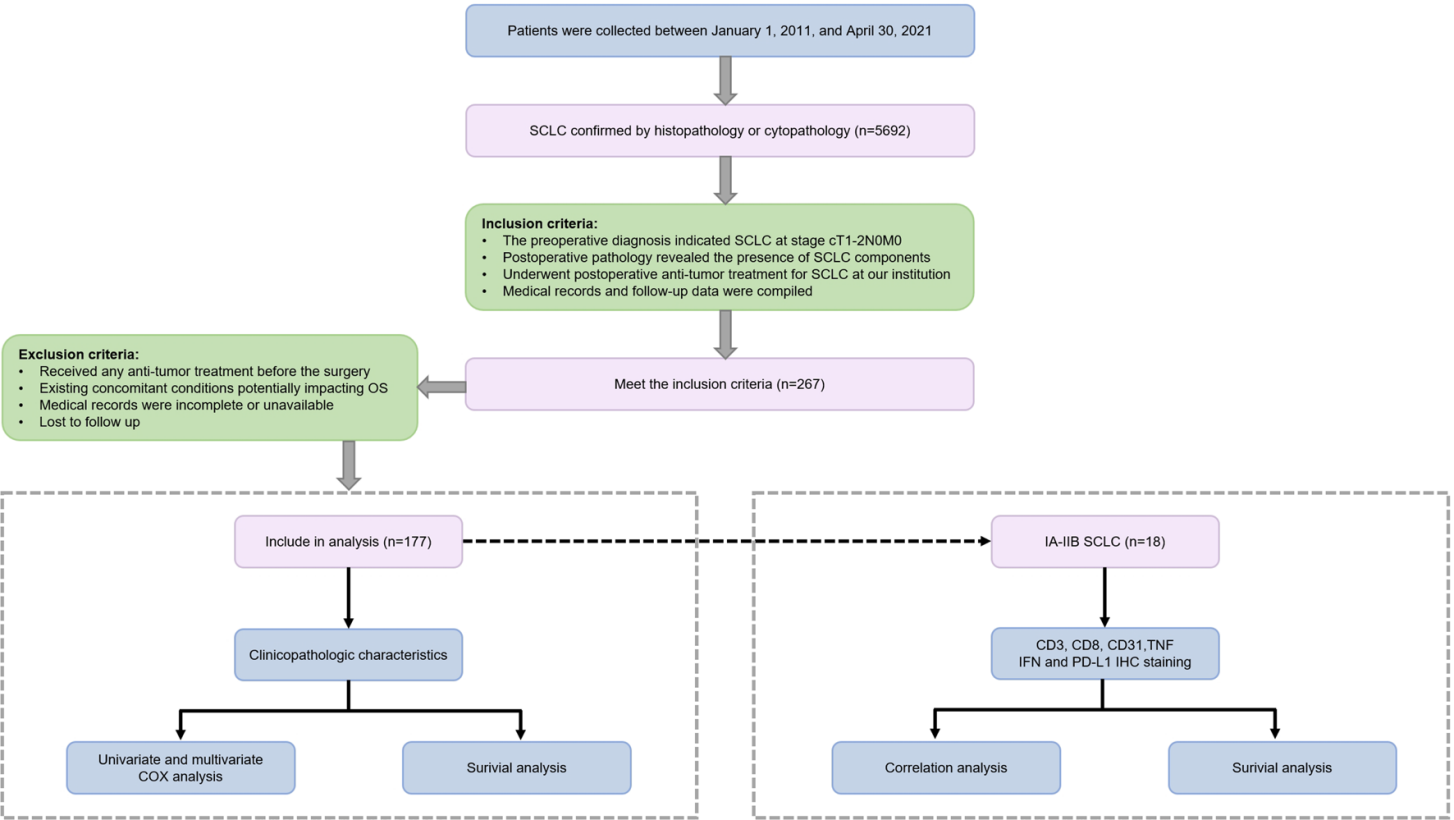
The screening process for patients and workflow of this study

The Ethics Committee of the First Affiliated Hospital of USTC approved the study. From January 1, 2011 to April 30, 2021, total of 5692 patients were diagnosed with SCLC through histopathological or cytopathological examination at the First Affiliated Hospital of USTC. The patients with SCLC were collected by consulting medical records provided by the Medical Records and Pathology Departments at the First Affiliated Hospital of the USTC. Subsequently, patients who underwent surgical resection and were pathologically diagnosed with SCLC were selected. Ultimately, our study included 177 patients with thorough follow-up and comprehensive medical records. The screening process for patients and workflow of this study is shown in Fig. 1. In this study, the majority of patients were followed up via telephone, while a small subset underwent follow-up appointments at outpatient clinics. The primary and secondary endpoints of were overall survival (OS) and progression-free survival (PFS) respectively. Follow-up deadline: December 2023.

### Inclusion criteria

1. Following initial suspicion of lung cancer from chest CT, positron emission tomography-computed tomography (PET-CT), and other imaging tests, the patients underwent a thorough evaluation, validating eligibility for curative surgery. Subsequent postoperative pathology examination definitively diagnosed SCLC.
2. Patients underwent biopsy procedures, including operation, fiber optic bronchoscopy, or endobronchial ultrasound-guided transbronchial needle aspiration (EBUS-TBNA)/mediastinoscopy, and pulmonary puncture guided by imaging for the evaluation of pulmonary lesions and/or mediastinal lymph nodes. While the initial biopsy pathology indicated non-small cell lung cancer (NSCLC), post-surgical pathology examination revealed the presence of SCLC components in the patients.
3. Patients diagnosed with SCLC using the preoperative diagnostic methods specified in criterion 2 must satisfy the clinical staging of cT1-2N0M0.
4. The patients underwent anti-tumor treatment for SCLC at the First Affiliated Hospital of USTC, which involved surgery, chemotherapy, and/or radiotherapy.
5. Both the medical records and follow-up data of the patients were complete.

### Exclusion criteria

1. The patients received any anti-tumor treatment before the surgery.
2. The patients presented with severe cardio-cerebrovascular disease or other conditions that could significantly influence the prognosis.
3. The patients were lost to follow-up, and medical records or crucial information were incomplete or unavailable.

### Postoperative adjuvant treatment cohort

According to current international clinical guidelines, postoperative adjuvant chemotherapy using platinum-based regimens is typically advised for patients with SCLC [2, 4]. In this study, patients who completed at least four cycles of postoperative chemotherapy, regardless of whether they received radiation, were categorized as having completed postoperative adjuvant therapy; those who did not meet this criterion were classified as having incomplete postoperative adjuvant therapy. Patients undergoing postoperative chest radiation typically received a dose ranging from 50 to 60 Gy, which was completed within a 30-day period. Patients subjected to prophylactic cranial irradiation (PCI) were administered 25 Gy in 10 fractions.

### IHC staining

The IHC staining was performed to assess the expression levels of CD3^+^, CD8^+^, CD31^+^, PD-L1^+^ cells within both tumor and stromal tissues of SCLC. In addition, IFN-γ and TNF-α antibodies were used in IHC staining of tumor tissues to assess the extent and intensity of positive reactions. Morphologically identified the components of inflammatory cells in tumor and tumor stroma tissues, and accurately assessed the proportion of inflammatory cells in the tumor and stroma. Briefly, paraffin-embedded sections were incubated in an oven for 3 h. Subsequently, the sections underwent dewaxing in xylene and ethanol (100%, 95%, 85%, and 75%, each for 5 min) and were placed in citric acid buffer for antigen retrieval. After being blocked with peroxide blocking solution for 30 min, slides were incubated overnight in a wet chamber at 4 °C with primary antibodies at concentrations of 1:150 (anti-CD3, KIT-0003, MXB Biotechnologies, Fuzhou, China), 1:1000 (anti-CD8, MAB-0021, MXB Biotechnologies, Fuzhou, China), 1:50 (anti-CD31, MAB-0021, MXB Biotechnologies, Fuzhou, China), 1:100 (anti-IFNɑ, ab9579, Abcam, MA, USA), and 1:500 (anti-TNFγ, ab9579, Abcam, MA, USA). Subsequently, the slides were incubated for 30 min using a secondary antibody (KIT-5020, MaxVision-HRP mouse/rabbit, Maxim, Fuzhou, China). The DAB kit (ZLI-9018, Zhongshan Golden Bridge, Beijing, China) was employed for section staining, followed by counterstaining with hematoxylin, dehydration, and mounting. Two pathologists, blinded to the clinicopathological data, independently scored all samples, and the mean count was utilized.

### IHC for PD-L1 by Dako

The paraffin tissue sections were incubated at 60 °C for 1 h, then loaded onto the Dako autostainer and treated with Proteinase K for 5 min. Subsequently, the Dako autostainer applied the antibodies (PD-L1, 22C3, Dako, Santa Clara, USA), and slides were incubated at room temperature for 30 min. Following a buffer wash, slides underwent a 30-min incubation with the labeled polymer, HRP, at room temperature. The DAB^+^ substrate-chromagen solution was then applied for 10 min. Counterstaining was carried out with Dako automation hematoxylin for 5 min, followed by post-counterstaining with a bluing agent for 1 min. After washing, slides were dehydrated in three sequential washes of 70%, 95%, and 100% reagent alcohol, and three xylenes baths before coverslips were applied. Binary scoring, utilizing the FDA-approved companion assay cut-point for PD-L1–stained tumor-infiltrating ICs (of any staining intensity, covering ≥ 1% of the tumor area), was conducted by two board-certified. Anatomic pathologists specialized in PD-L1 interpretation. In cases of discordance, a consensus-based final PD-L1 score was assigned after re-review.

### Statistical analysis

OS was defined as the duration from surgery to death from any cause or the last follow-up. Kaplan–Meier method assessed OS, and the log-rank test gauged survival differences. The Cox proportional hazard model identified independent prognostic factors for OS in surgically resected SCLC patients. In the multivariate model, all factors with a *p*-value < 0.05 in the univariate analysis were included. Bilateral tests were conducted, and statistical significance was set at *p* < 0.05. The analyses and graphs were performed using R language (version 4.1.3) packages, including survminer (version 0.4.8), survival (version 3.2.12), ezcox (version 1.0.2), forestplot (version 2.1.0), and tableone (version 0.13.2).

## Results

### Surgical resection rate of SCLC patients

From January 1, 2011, to April 30, 2021, a cumulative total of 5692 individuals were diagnosed with SCLC through cytological or histopathological examination at the First Affiliated Hospital of the USTC. A total of 279 patients who underwent surgical resection and received a pathological diagnosis of SCLC, yielding a surgical resection rate of 4.90% for SCLC at our institution over the period from 2011 to 2021. After thoroughly reviewing medical records and follow-up data, the study identified a cohort of 177 patients who ultimately met the inclusion and exclusion criteria (Supplementary Table 1).

### Patients characteristics

Clinicopathological features of the 177 surgically resected SCLC patients is shown in Table 1. Among the 177 SCLC patients undergoing surgical treatment in this study, comprising 143 males (80.79%) and 34 females (19.21%), with a median age of 65 years (range: 35 to 84). In accordance with the World Health Organization (WHO) definition, individuals who have never smoked or consumed fewer than 100 cigarettes in their lifetime are classified as “Never smokers” while those who have exceeded a cumulative history of 100 cigarettes are classified as “Ever smokers” [13]. Of the total cohort, 96 patients (54.24%) were divided into “Ever smokers”, whereas 81 patients (45.76%) were categorized as “Never smokers”, with 33 cases (18.64%) involving female patients. Most patients (109, 61.58%) were free from additional comorbidities, whereas 68 cases (38.42%) presented with concurrent chronic conditions, with hypertension being the most common (36, 20.34%), followed by type 2 diabetes (13, 7.34%), and coronary heart disease (10, 5.65%). Among all patients, 58 (32.77%), 12 (6.78%), 8 (4.52%), 31 (17.51%), 52 (29.38%), and 16 (9.04%) were diagnosed with clinical stages IA, IB, IIA, IIB, IIIA, and IIIB, respectively, according to the surgical excision specimen pathology results. Of all these patients, 86 (48.59%), 54 (30.51%), 21 (11.86%), and 16 (9.04%) patients with pathological stage pathological T staging (pT1), pT2, pT3 and pT4, respectively. Regarding pathological N staging (pN), the predominant proportion of patients exhibited no lymph node metastasis (pN0), constituting 90 cases (50.85%). Patients classified as pN1 and pN2 staging amounted to 34 cases (19.21%) and 53 cases (29.94%), respectively. The range of surgically resected lymph nodes varied between 1 and 48, with an average of 16.3 lymph nodes per patient undergoing pathological examination. Among the 34 patients with pN1 staging, the predominant site for metastatic lymph nodes was the level 10 lymph nodes, accounting for 31 cases (91.18%). Thirty-three and twenty-three patients respectively received a pN2 stage diagnosis due to tumor metastasis to the level 7 and level 4 lymph nodes (62.26% and 43.40% respectively). In this study, these sites represented the most frequent locations of lymph node metastasis among patients with pN2 staging (Supplementary Table 2). Overall, 169 patients (95.48%) underwent pulmonary lobectomy, and 2 patients (1.13%) were executed segmental lung resection. Additionally, 6 patients (3.39%) underwent unilateral pneumonectomy. Patients with tumors located in the right lung (91, 51.41%) were slightly more prevalent than those in the left lung (86, 48.59%). In postoperative pathological analysis, bronchial cancer embolus were identified in 8 cases, constituting 4.52% of the total. The overall R0 surgical resection rate achieved 97.18%. Moreover, 29 cases (16.38%) manifested a combination of pathological components, with the preponderance (24 cases, 13.56%) concurrently presenting pulmonary adenocarcinoma components. In this study, patients who undergo at least four cycles of adjuvant chemotherapy with or without radiotherapy after surgery are defined as the completed postoperative adjuvant treatment group; otherwise, they are categorized as the incomplete group. Postoperatively, 97 cases (54.80%) of patients received at least four cycles of adjuvant chemotherapy, while an additional 21 (11.86%) patients did not complete postoperative adjuvant treatment consisting of at least four cycles of chemotherapy. One-third (59, 33.33%) of patients did not undergo any postoperative treatment, citing either personal preferences or intolerance to chemotherapy or radiotherapy side effects.

**Table 1:** Clinicopathological features of the 177 surgically resected SCLC patients

Clinical characteristics	SCLC (n = 177) (%)
*Gender*	
Female	34 (19.21)
Male	143 (80.79)
*Age*	
> 65	90 (50.85)
≤ 65	87 (49.15)
*Smoking history*	
Never smoker	81 (45.76)
Ever smoker	96 (54.24)
*Concomitant diseases*	
No	68 (38.42)
Yes	109 (61.58)
*Pathological T stage*	
pT1a	7 (3.95)
pT1b	39 (22.03)
pT1c	40 (22.60)
pT2a	30 (16.95)
pT2b	24 (13.56)
pT3	21 (11.86)
pT4	16 (9.04)
*Pathological N stage*	
pN0	90 (50.85)
pN1	34 (19.21)
pN2	53 (29.94)
*TNM stage*	
IA	58 (32.77)
IB	12 (6.78)
IIA	8 (4.52)
IIB	31 (17.51)
IIIA	52 (29.38)
IIIB	16 (9.04)
*Extent of resection*	
Lobectomy	169 (95.48)
Pneumonectomy	2 (1.13)
Segmentectomy	6 (3.39)
*Tumor localization*	
Left lung	86 (48.59)
Right lung	91 (51.41)
*Pathological subtype*	
Combine	29 (16.38)
Pure	148 (83.62)
*R0 resection*	
No	5 (2.82)
Yes	172 (97.18)
*Endobronchial cancer thrombus*	
No	169 (95.48)
Yes	8 (4.52)
*Postoperative therapy*	
Complete	80 (45.20)
Incomplete	97 (54.80)

Values are presented as n (%)

### Univariate and multivariate analysis for surgically resected SCLC patients

**Fig. 2:**
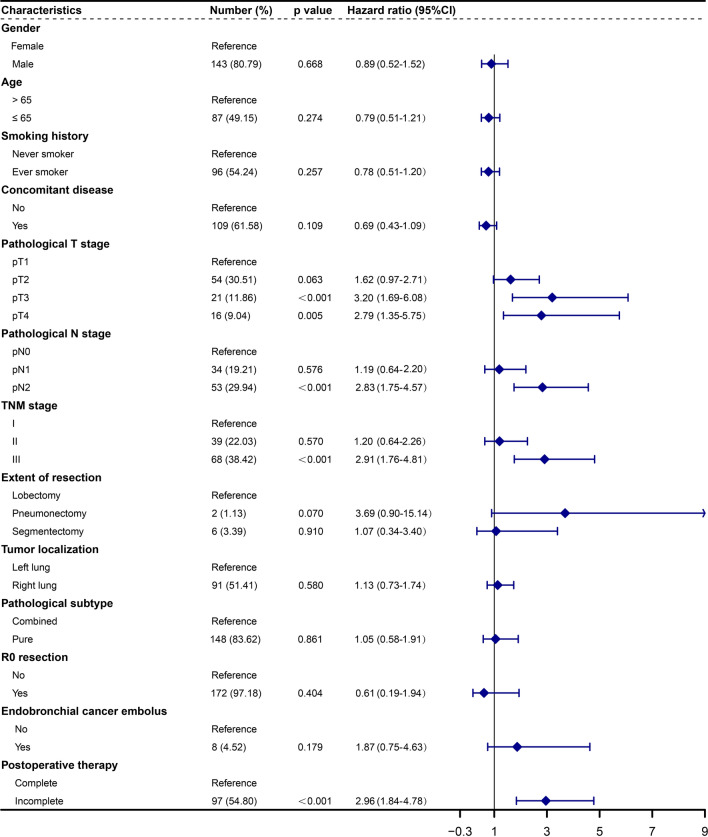
Univariate analysis of factors associated with OS for the 177 surgically resected SCLC patients

**Fig. 3:**
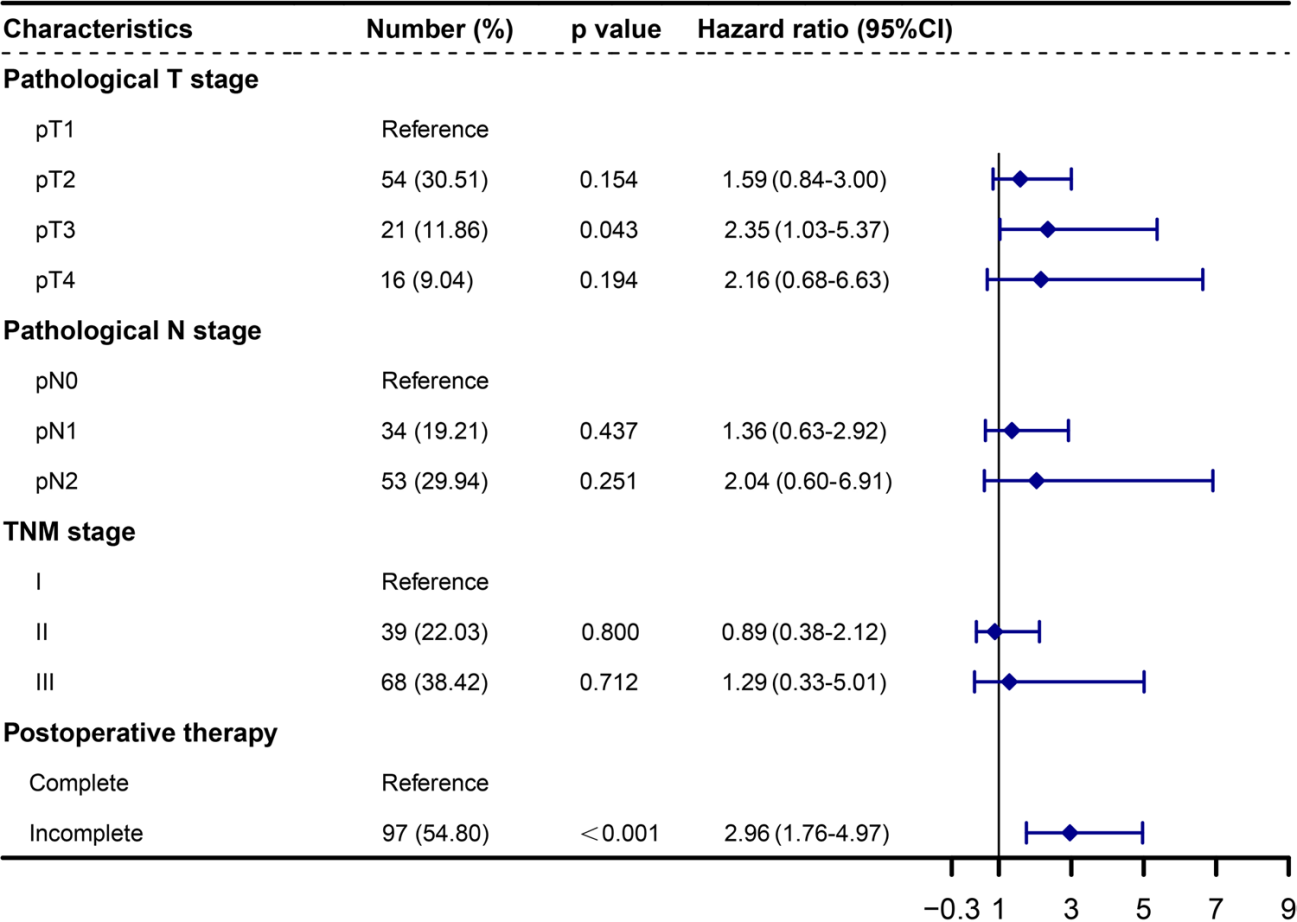
Multivariate analysis of factors associated with OS for the 177 surgically resected SCLC patients

The median follow-up time was 49.71 years (95%CI 43.05–56.38), and the median OS was 46.67 months (95%CI 41.84–51.51). Patients were stratified according to clinical and pathological features, including gender, age, smoking history, comorbidities, pT, pN, TNM staging, extent of resection, tumor localization, pathological subtype, R0 resection, endobronchial cancer embolus and postoperative adjuvant treatment. Univariate and multivariate Cox analysis were subsequently performed. In the univariate analysis, statistically significant differences were observed among cohorts stratified based on pT, pN, TNM staging, and postoperative adjuvant treatment. For the entire cohort, the univariate analysis indicated that patients with pT3-4, pN2, and stage III were associated with a worse prognosis when compared to patients with pT1, pN0, and stage I (*p* < 0.001, HR 3.20, 95%CI 1.69–6.08; *p* = 0.005, HR 2.79, 95%CI 1.35–5.75; *p* < 0.001, HR 2.83, 95%CI 1.75–5.47; *p* < 0.001, HR 2.91, 95%CI 1.76–4.81, respectively). Meanwhile, patients who did not receive complete postoperative adjuvant therapy exhibited poorer survival (*p* < 0.001, HR 2.96, 95%CI 1.84–4.78) (Fig. 2). Upon integrating factors showing significant differences in the univariate analysis into the multivariate analysis, it was disclosed that pT3 and incomplete postoperative adjuvant treatment emerged as adverse prognostic factors following surgical resection of SCLC (*p* = 0.043, HR 2.35, 95%CI 1.03–5.37; *p* < 0.001, HR 2.96, 95%CI 1.76–4.97, respectively) (Figs. 2, 3).

### Survival analysis for surgically resected SCLC patients

**Fig. 4:**
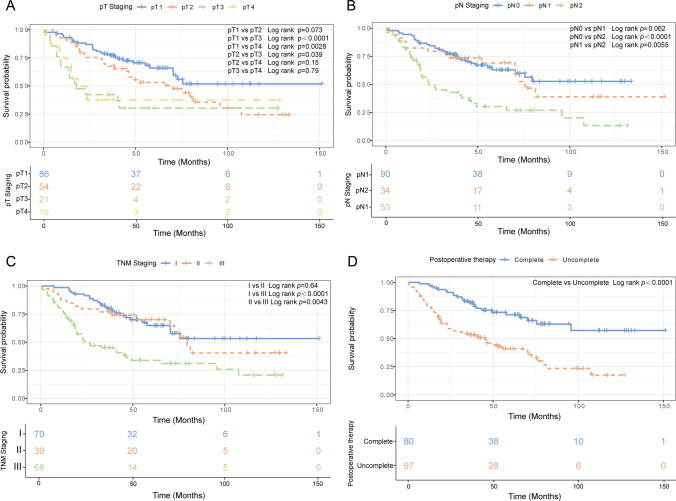
Overall survival of different pathological T, N, and TNM stage in the 177 surgical resection SCLC patients

In survival analysis, the OS of patients with pT1 tumors exhibited a noteworthy survival advantage compared to those with pT3 and pT4 tumors (*p* < 0.0001 and 0.0028, respectively); conversely, the OS of pT2 patients fails to achieve statistical significance when compared to those with pT1 (*p* = 0.073) (Fig. 4A). Patients classified as pN0 demonstrate a notably superior OS compared to those categorized as pN2 (*p* < 0.0001), and the OS of patients with pN1 designation significantly surpasses that of counterparts with pN2 status (*p* = 0.0055). Notably, the observed difference in OS between patients designated as pN0 and pN1 did not attain statistical significance (*p* = 0.062) (Fig. 4B). The OS among patients with I and II staging exhibited a marked superiority compared to those with III staging (*p* < 0.0001, *p* = 0.0043, respectively); however, there was no statistically significant distinction in OS between patients at stages I and II (*p* = 0.64) (Fig. 4C). Consistent with the results of Cox analysis, patients who completed postoperative adjuvant treatment (OS: 6.87–151.17 months, median OS: 49.13 months) demonstrated markedly enhanced survival compared to those who did not finish adjuvant treatment (OS: 0.53–126.97 months, median OS: 34.47 months) (*p* < 0.0001) (Fig. 4D).

### IHC analysis of 18 surgically resected SCLC patients with IA-IIA staging

We considered selecting a subset of pathological specimens from the 177 surgically resected SCLC patients for IHC staining of selected indicators in order to explore potential prognostic indicators for surgically resected SCLC. Since the analysis in the preceding section demonstrated no statistically significant difference in survival rates between stage I and II patients; however, there was a statistically significant difference in survival rates between patients who received complete and incomplete postoperative adjuvant therapy, and current clinical guidelines recommend surgical treatment for stage I-IIA (cT1-2N0M0) SCLC patients. Hence, this study focused only on conducting IHC analysis on 18 patients who underwent complete postoperative adjuvant therapy within this stage range. Due to the disparate distribution of patient across stages IA, IB, and IIA (30, 5, and 5, respectively) patients with complete postoperative adjuvant therapy, we utilized stratified randomization based on the distribution of patients within each stage (75.0%, 12.5%, and 12.5%, respectively). From the 40 cases of stage IA-IIB patients who underwent complete postoperative adjuvant therapy, 14, 2, and 2 patients at stages IA, IIB, and IIA, respectively, were randomly chosen. Subsequently, we performed IHC staining for T cells and markers related to tumor immune or angiogenesis function, including CD3, CD8, CD31, IFN-γ, TNF-α, and PD-L1. This analysis was designed to evaluate the influence of inflammatory cell levels in both the tumor and its stroma on postoperative SD and PD in patients. Initially, distinguish the regions and proportions occupied by both the tumor and its stroma in each pathological formalin-fixed paraffin-embedded (FFPE) specimen through HE staining. Subsequently, IHC staining was conducted on the FFPE specimens using CD3 and CD8 antibodies to selectively label T cells and cytotoxic T lymphocytes (CTLs). Following the staining, the proportions of inflammatory cells, CD3^+^, and CD8^+^ cells within both the tumor and its stroma were quantified individually (Supplementary Table 3).

In order to address the influence of variable proportions of tumor and tumor stroma components in individual FFPE specimens on the analysis results, we establish the inflammation cell score as the product of the percentage it comprises in the tumor or tumor stroma and the percentage of tumor or tumor stroma in the sample. Similarly, the assessment of CD3^+^ or CD8^+^ cells was ascertained by multiplying the percentage they represent in the inflammatory cells of the tumor or tumor stroma by the score of inflammatory cells in the respective sample's tumor or tumor stroma.

### Proportion of tumor and stroma inflammatory cells in surgically resected SCLC patients with postoperative SD and PD

**Fig. 5:**
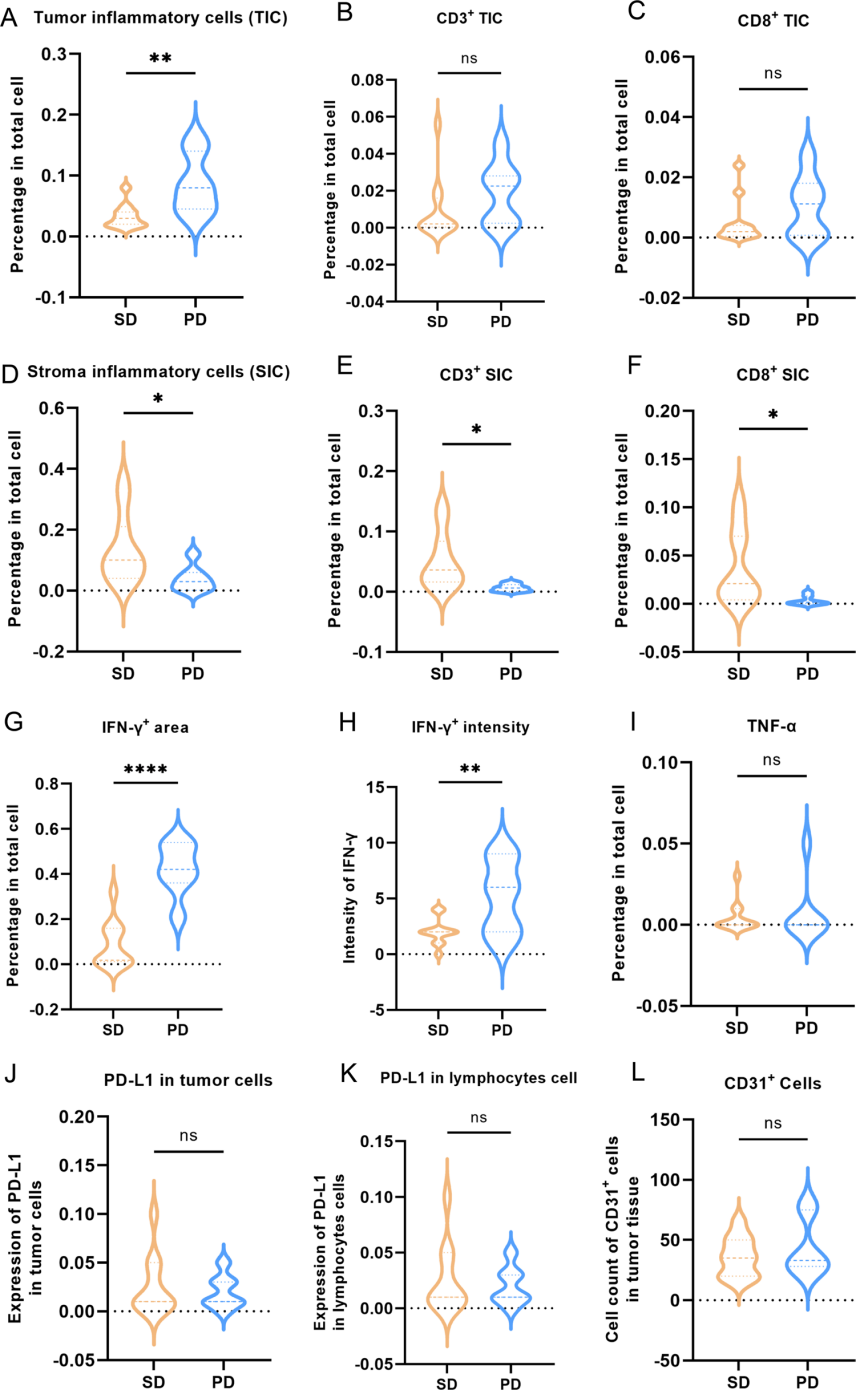
Violin plot based on the IHC staining results of the levels of TIC, SIC, as well as IFN-γ^+^, PD-L1^+^, CD3^+^, CD8^+^, and CD31^+^ cells within both postoperative SD and PD patients

The IHC analysis results revealed a significant decline in the score of tumor inflammatory cells (TIC) in patients who achieved postoperative SD compared to those who suffered postoperative PD. (*p* = 0.0047) (Fig. 5A). There was a trend of elevated levels in CD3^+^ or CD8^+^ TIC in patients with postoperative PD, but it had not reached statistical significance (*p* = 0.2596 and 0.2184, respectively) (Fig. 5B, C). In contrast, the score of stroma inflammatory cells (SIC) was higher in patients with postoperative SD compared to those experiencing PD (*p* = 0.0453) (Fig. 5D). Similarly, notable increases in the CD3^+^ or CD8^+^ SIC levels were observed in patients with postoperative SD (*p* = 0.0262 and 0.0330, respectively) (Fig. 5E, F). In addition, patients who achieved postoperative SD exhibited lower levels of IFN-γ expression in both area and intensity (*p* < 0.0001 and 0.0091, respectively) compared to those who suffered postoperative PD (Fig. 5G, H).

### Expression levels of TNF-ɑ, PD-L1 and CD31 in surgically resected SCLC patients with postoperative SD and PD

TNF-α was identified in the tumor tissues of three patients with postoperative SD and one patient with postoperative PD using IHC staining, and there was no statistically significant disparity in TNF-α levels between the two groups (*p* = 0.7007) (Fig. 5I). The IHC staining revealed positive PD-L1 expression in tumor cells of only one patient (mild expression, 3%), and in lymphocytes of six patients with postoperative SD and three patients with postoperative PD (mild expression, ranging from 3 to 5%). However, no significant difference was observed in PD-L1 expression in tumor and lymphocytes cells between patients who achieved postoperative SD and those who experienced postoperative PD (*p* = 0.6738, 0.9238, respectively) (Fig. 5J, K). Overall, the IHC findings in this study suggest a notably low expression of PD-L1 in SCLC. Additionally, in the IHC results of all 18 patients, the CD31^+^ cell counts within the samples varied from 15 to 80. There was no statistically significant difference in the counts of CD31^+^ cell between the cohorts of patients with postoperative SD and PD (p = 0.4504) (Fig. 5L).

### Prognostic value of TIC, SIC, and IFN-γ levels on postoperative prognosis

**Fig. 6:**
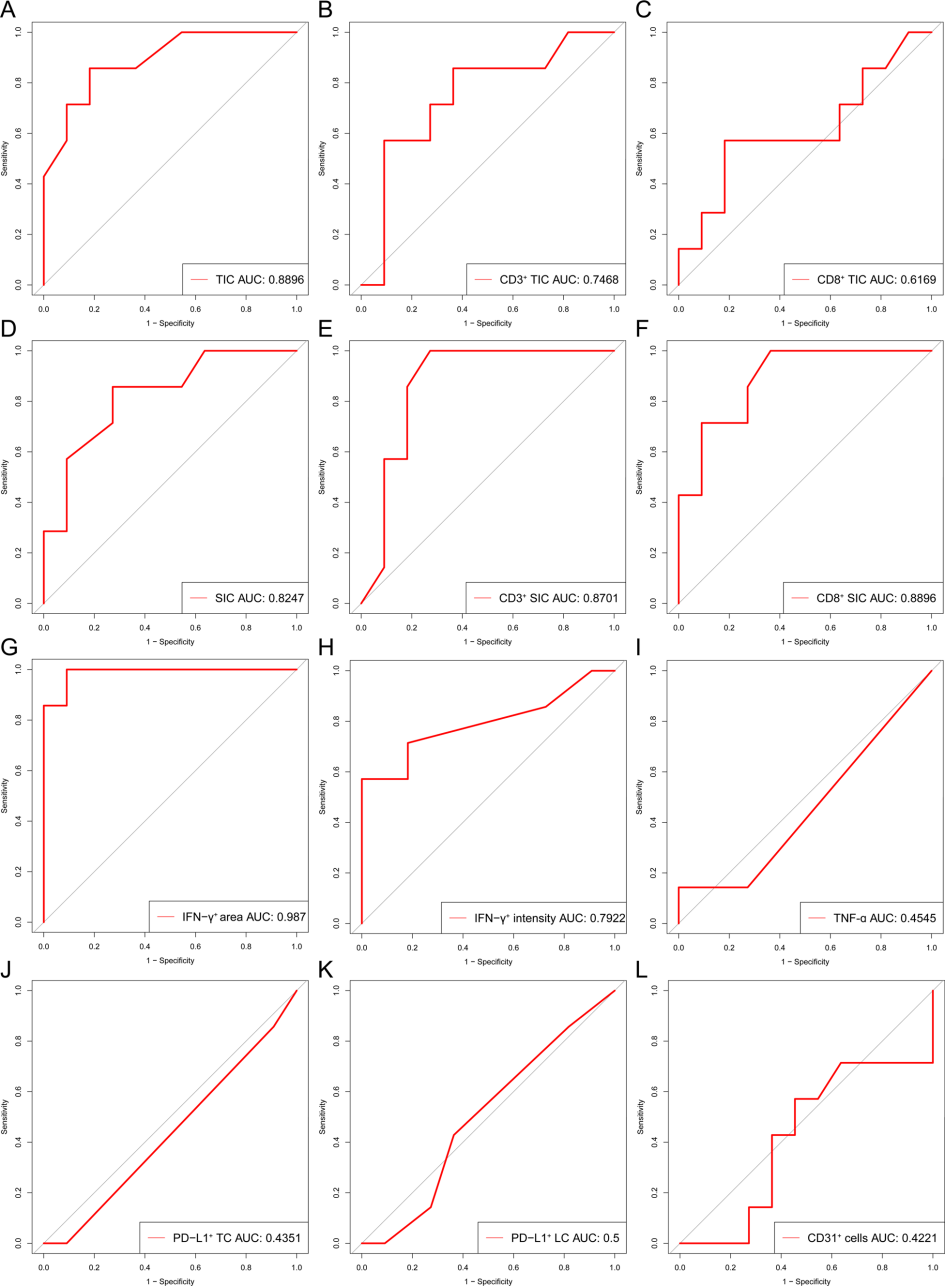
The specificity and sensitivity of the markers including TIC, SIC, as well as IFN-γ^+^, PD-L1^+^, CD3^+^, CD8^+^, and CD31^+^ cells for distinguishing postoperative SD and PD patients were revealed by ROC curves

The receiver operating characteristic (ROC) curve demonstrates that the prognostic capability of TIC levels in for postoperative SD and PD yields an area under the curve (AUC) of 0.8896 (Fig. 6A). The predictive performance of CD3^+^ and CD8^+^ TIC levels for postoperative prediction of SD and PD was lower than that of overall TIC levels, with AUC values of 0.7468 and 0.6169, respectively (Fig. 6B, C). The predictive values of SIC levels for postoperative SD and PD achieve AUC of 0.8247 (Fig. 6D). Similarly, the levels of CD3^+^ and CD8^+^ SIC demonstrate predictive efficacy for postoperative PD and SD similar to the SIC levels (AUC = 0.8701, 0.8896, respectively) (Fig. 6E, F).Moreover, The ROC curve exhibits outstanding discriminatory capability among patients experiencing postoperative SD and PD. This discrimination relies on the assessment of the area and intensity of IFN-γ^+^ cells within tumor tissues, yielding corresponding AUC values of 0.9870 and 0.7922 (Fig. 6G, H). In this study, the predictive value of IFN-γ, TNF-α, PD-L1, and CD31 for postoperative prognosis was not satisfactory, with corresponding AUC values of 0.7922, 0.4545, 0.4351, 0.5, and 0.4221, respectively (Fig. 6I–L).

**Fig. 7:**
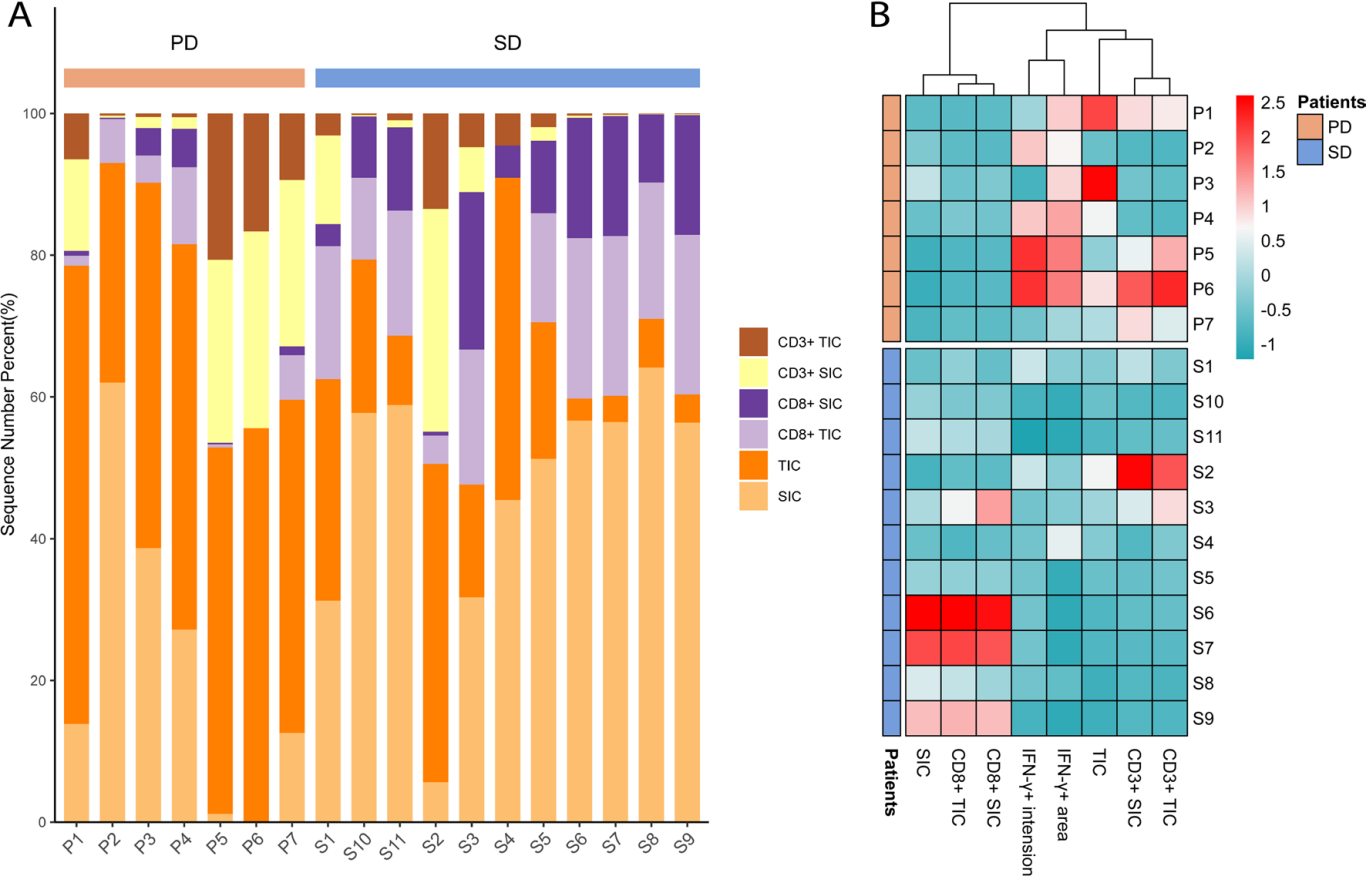
Bar graph and heatmap based on the levels of TIC, SIC, CD3^+^ and CD8^+^ cells of the 18 surgical resection SCLC patients. **A** Bar charts exhibited the proportions of TIC, SIC, CD3^+^ and CD8^+^ cells within each sample of postoperative SD and PD patients. **B** Clustered heatmap based on Z-score normalized data representing TIC, SIC, CD3^+^, and CD8^+^ cell counts

**Fig. 8:**
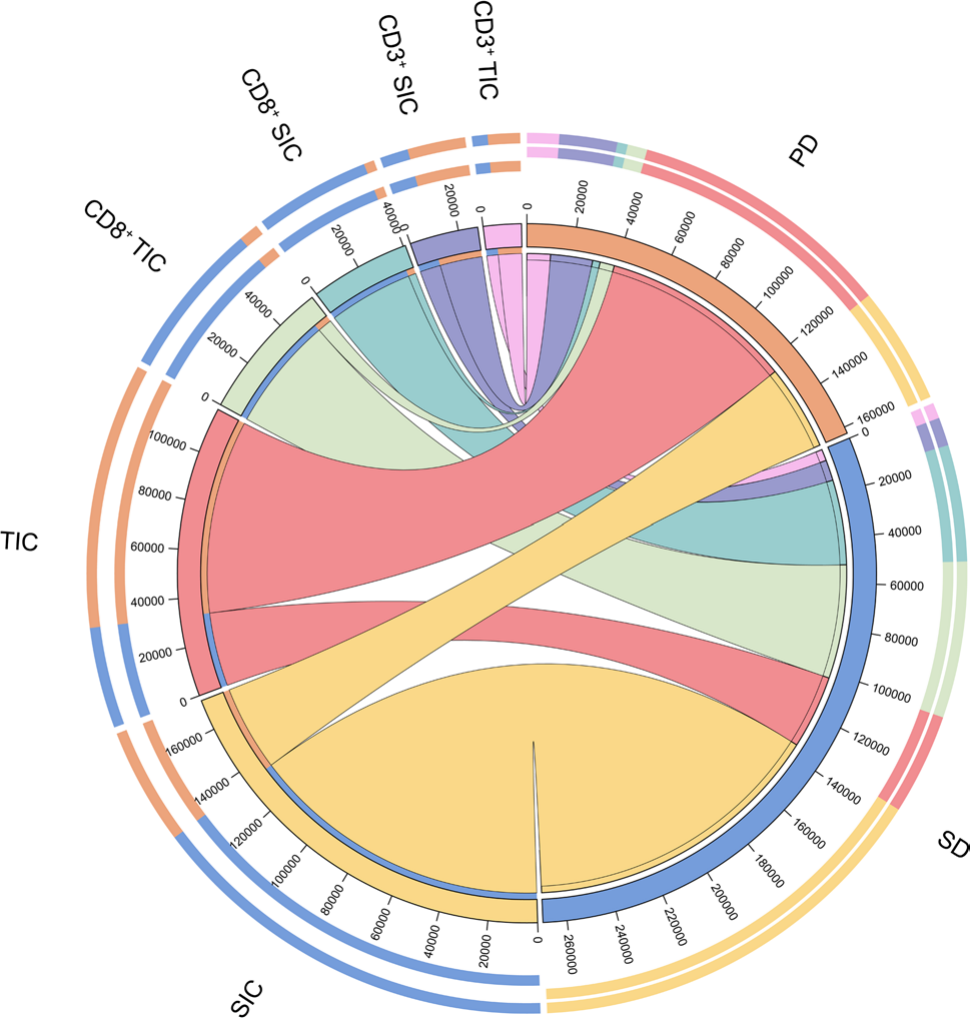
Circos diagram built upon the data of the levels of SIC, TIC, CD3^+^, and CD8^+^ cells in postoperative SD and PD patients samples

Generate bar graph based on the proportions of TIC, SIC, CD3^+^ and CD8^+^ cells within each sample of patients. The bar graph demonstrates elevated levels of SIC, CD8^+^ SIC, and CD8^+^ TIC in patients with postoperative SD, while TIC, CD3^+^ SIC, and CD3^+^ TIC levels significantly increase in patients with postoperative PD (Fig. 7A). We subsequently created a clustered heatmap using Z-score normalized data to depict the enrichment levels of various indicators in patients with postoperative SD and PD groups. The relationship revealed from heatmap clustering and enrichment showed upregulated levels of SIC and CD8^+^ cells in patients with postoperative SD, while those with postoperative PD exhibited elevated levels of TIC, IFN-γ, and CD3^+^ cells (Fig. 7B). Circos diagram have an advantage in representing the relative proportions and percentages, emphasizing the importance of different categories or components in the whole [14]. Therefore, we also utilized Circos diagram to depict the proportions of TIC, SIC, CD3^+^, and CD8^+^ cells in each sample, illustrating the relationships of these indicators between postoperative SD and PD patients. The Circos diagram visualizes a strong association between SIC and CD8^+^ cells with postoperative SD status, and TIC and CD3^+^ cells with postoperative PD status (Fig. 8).

**Fig. 9:**
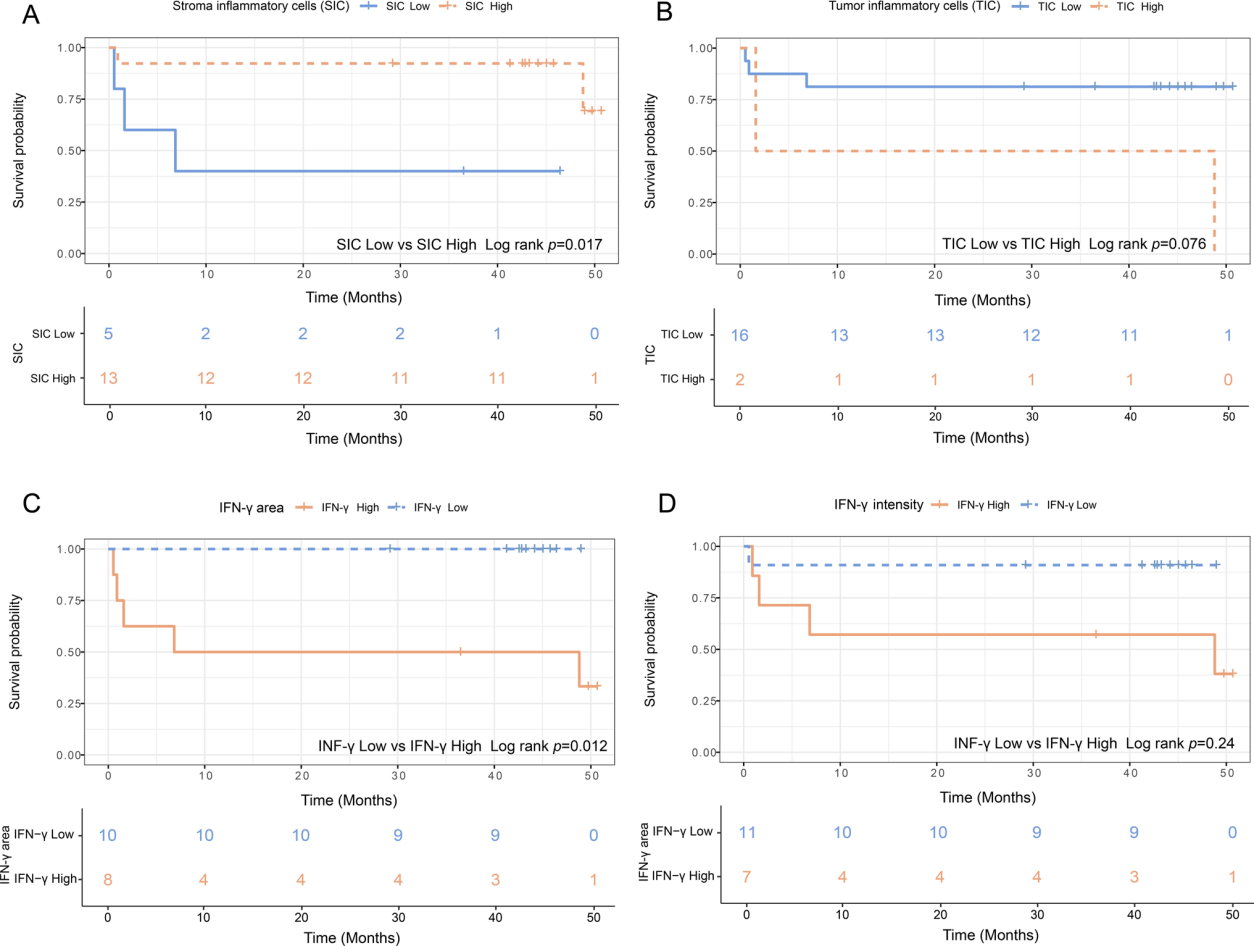
Survival analysis of SIC, TIC, and IFN-γ levels on postoperative prognosis. **A** Survival analysis for patients categorized into SIC high and low groups based on the optimal cut-off value of 0.09. **B** Survival analysis for patients categorized into TIC high and low groups based on the optimal cut-off value of 0.04. **C** Survival analysis for patients categorized into IFN-γ area high and low groups based on the optimal cut-off value of 0.16. **D** Survival analysis for patients categorized into IFN-γ intensity high and low groups based on the optimal cut-off value of 2

Optimal cut-off values were utilized to categorize the 18 patients into high SIC and TIC groups as well as low SIC and TIC groups, using cut-off values of 0.09 and 0.04, respectively. Additionally, for the area and intensity of IFN-γ^+^ cells, the optimal cut-off values were determined as 0.16 and 2, respectively. Survival analysis revealed a positive correlation between raised levels of SIC and IFN-γ^+^ cell area and higher survival rates (*p* = 0.017, 0.012, respectively) (Fig. 9A, B). Patients with high levels of TIC and positive IFN-γ^+^ intensity also appeared to have better survival, although statistical significance was not reached in both cases (*p* = 0.076, 0.24, respectively) (Fig. 9C, D).

## Discussion

SCLC has two staging systems, namely the TNM staging system proposed jointly by the IARC and the American Joint Committee on Cancer (AJCC), and the VALSG staging system proposed by the Veterans Administration Lung Study Group (VALSG) [15, 16]. The VALSG staging system remains extensively utilized in clinical diagnosis, treatment, and the design of clinical trials. This was chiefly attributed to the critical role of radiotherapy in managing SCLC and the heightened effectiveness of this staging system in directing the selection of treatment plans [17]. Concurrent radiochemotherapy is mainly applied to limited-stage SCLC (LS-SCLC), while systemic chemotherapy or chemoimmunotherapy is more suitable for extensive-stage SCLC (ES-SCLC). In clinical practice, the staging of SCLC is typically performed using the TNM and VALSG staging systems. According to the eighth edition of the AJCC TNM staging system, clinical stages I–III are typically associated with LS-SCLC, while clinical stage IV corresponds to ES-SCLC in the VALSG staging system. Specifically, patients in stages I, II, and III constituted 4%, 1%, and 25%, respectively, whereas stage IV represented a substantial majority at 70% [15, 18]. As per the current guidelines of the American Society of Clinical Oncology (ASCO), National Comprehensive Cancer Network (NCCN) and the European Society for Medical Oncology (ESMO), surgery is recommended exclusively for SCLC patients with stages I-IIA (T1-2N0M0) [2, 19, 20]. Although less than 5% of all SCLC cases are eligible for surgical treatment, this subgroup of patients could be regarded as potential candidates for a more favorable prognosis post-surgical intervention. Moreover, before surgery, it is recommended for all patients to undergo a pathological assessment of mediastinal lymph nodes to detect and eliminate potential occult lymph node metastasis. Diagnostic methods may include mediastinoscopy, EBUS-TBNA, endoscopic ultrasound-guided fine needle aspiration (EUS-FNA), or thoracoscopy [2, 19, 20]. Nevertheless, the staging of many SCLC patients undergoing surgical resection is often underestimated due to the highly malignant nature and the propensity for metastasis, especially in lymph node assessment. Our observation revealed that certain SCLC patients, following surgical resection, had a more favorable prognosis than anticipated. Therefore, a review was conducted on patients undergoing surgical resection to reassess the role of surgery in treating SCLC.

Previous studies emphasized the role and potential benefits of surgical intervention for patients with specific stages of SCLC. Patients with I ~ IIA stage SCLC may derive benefits from surgery. Existing data indicate that the 5-year survival rates for the surgical and non-surgical groups range between 27 to 73% and 4 to 44%, respectively. Yang and colleagues, utilizing propensity score matching analysis with the national cancer database (NCDB) database, discovered a significant improvement in the 5-year survival rate due to surgery (47.6% vs. 29.8%, *p* < 0.01) [11]. Regarding surgical approaches, several retrospective studies and subgroup analyses within meta-analysis consistently demonstrate that survival outcomes was superior in the lung lobe resection group compared to the wedge resection group [11, 21]. The role of surgery in stage IIB ~ IIIA SCLC remains a subject of controversy. Despite positive outcomes in retrospective studies, the observed median survival range (17–31.7 months) did not demonstrate a significant enhancement when compared to the CONVERT study, which reported a median survival of 25 months with synchronous chemoradiotherapy [22]. Consequently, the efficacy of surgery in stages IIB ~ IIIA SCLC and its applicability to specific subgroups remain subjects of debate. For stage IIIB-IIIC SCLC, there is a lack of compelling evidence supporting the effectiveness of surgery; hence, surgery is not recommended as a treatment option.

Over the decade spanning 2011 to 2021, our institution identified 5692 individuals with SCLC through comprehensive pathological assessments. Of this cohort, 279 patients underwent surgical intervention, yielding a surgical resection rate of 4.90%, consistent with observations in other research and extensive epidemiological studies on SCLC. Among the cohort of 177 patients ultimately included in the study, 68 patients were identified with postoperative stage III, representing a substantial percentage of 38.42%. The outcomes of survival analysis indicate that, despite timely surgical resection, the OS of stage III patients persisted as lower than that of stage I and II patients, and there was no notable disparity in OS between stage I and II patients. Similarly, pN2 patients had a notably worse prognosis compared to pN0 and pN1 patients. Interestingly, no difference in survival was observed between pN0 and pN1 patients in this study cohort. Postoperative staging for SCLC patients in stage III frequently surpasses the preoperative clinical staging, primarily attributed to lymph node metastasis identified through pathological examinations, which remains imperceptible in imageological diagnosis. Studies indicated that in the context of SCLC, PET/CT emerges as a more effective staging modality than conventional imaging. Due to the significant correlation between the number and quality of lymph node dissection and postoperative lymph node staging, choice of postoperative treatment (e.g., the necessity for synchronous/sequential radiotherapy), and prognosis in SCLC patients, we detailedly recorded the surgically resected lymph node status of all patients in this study. In this study, an average of 16.3 lymph nodes were surgically removed per patient. Thorough lymph node dissection and standardized pathological examination further enhanced the reliability of the study findings to some extent. Our study revealed that lymph nodes at levels 10, 7, and 4 were the most frequent sites of metastasis. This emphasizes the importance of meticulous examination of lymph nodes in these regions before surgery. A previous study highlight a transition of 19% of patients initially categorized as LS-SCLC to ES-SCLC, and a reclassification of 8% of ES-SCLC cases as LS-SCLC after undergoing PET/CT assessment [23]. Patients with pT3-4 show a distinct survival disadvantage post-surgery compared to those with pT1-2. However, our observations reveal those patients with pN0-1 frequently present with a more favorable pT stage. Between 4 and 12% of SCLC patients exhibit only peripheral isolated nodules, posing challenges in distinguishing SCLC from NSCLC via chest imaging without percutaneous lung biopsy. The findings reiterate the crucial role of preoperative clinical staging assessments for patients, highlighting the importance of evaluating potential lymph node metastasis using techniques like mediastinoscopy or EBUS-TBNA. Our analysis revealed that a majority of patients in our cohort did not undergo PET/CT or EBUS-TBNA to assess N staging, potentially impacting the evaluation of surgical indications. To a certain degree, it also demonstrates the potential benefits of surgical treatment for SCLC patients with pT1-2N1M0 staging.

Li et al. revealed improved survival rates associated with adjuvant chemotherapy in patients lacking pathologic lymph node metastasis, whereas adjuvant chemoradiotherapy emerged as a potential factor of significant survival benefits for those with such metastasis [24]. Ye and colleagues conducted a retrospective cohort study utilizing the SEER database. Their findings suggested that among stage I and II SCLC patients, those who underwent surgery combined with chemotherapy exhibited a longer OS compared to those who underwent surgery alone [25]. Other pertinent studies have consistently shown the survival advantages associated with adjuvant chemotherapy in patients with resected LS-SCLC [26–32]. In the present study, univariate and multivariate analysis both indicate a correlation between incomplete postoperative adjuvant treatment and a poorer prognosis. Survival analysis reveals that patients who receive incomplete postoperative adjuvant therapy have a significantly worse prognosis compared to those in the complete adjuvant therapy group. These findings underscore the critical role of comprehensive treatment, particularly complete postoperative adjuvant therapy, for SCLC patients undergoing surgical resection. Hence, identifying suitable patients for resectable SCLC in conjunction with complete postoperative adjuvant therapy remains a pivotal factors in improving prognosis.

Rigorous investigations have substantiated that SCLC exhibits pronounced heterogeneity, spanning the domains of molecular genetics, histopathology, and tumor biology, constituting an inherent determinant of prognostic variations among patients with SCLC [33–35]. It is essential to identify biomarkers capable of precisely categorizing SCLC patients into subtypes, facilitating the development of more efficacious treatment strategies and evaluations of prognosis. Although multiple studies have indicated that certain genetic alterations drive the highly invasive and drug-resistant phenotypes of SCLC, including MYC, SLFN11, PTEN, and Notch/Sting pathways, genomic analysis of SCLC patients has yet to reveal a subtype defined by specific genetic changes [36–41]. However, most current studies focus on advanced-stage SCLC, and there is limited research on biomarkers for predicting postoperative survival in early-stage surgically resected SCLC. Byers and colleagues conducted a study on a cohort predominantly consisting of surgically resected LS-SCLC. They identified subgroups characterized by differential expression of transcription factors ASCL1, NEUROD1, and POU2F3, or low expression of these factors accompanied by inflammation gene expression. This classification stratifies SCLC into four distinct subtypes: SCLC-A, N, P, and I, each exhibiting unique therapeutic vulnerabilities. The study highlights the identification of SCLC-I as a new inflammatory subtype within SCLC, revealing those tumors falling under the SCLC-I category experience more pronounced benefits from immune checkpoint blockade. SCLC-I stands out from its subtypes primarily due to its prominent immune infiltration, characterized by the highest overall levels, and a notable elevation in the absolute counts of diverse immune cell populations, encompassing T cells, NK cells, and macrophages [42]. Utilizing IHC data from surgical samples of 18 patients diagnosed with stage pIA1-IIA SCLC in this investigation, we observed that individuals experiencing postoperative PD exhibited elevated levels of tumor-infiltrating inflammatory cells, whereas those with postoperative SD displayed increased inflammatory cell infiltration within the tumor stroma. Similarly, patients achieving postoperative SD demonstrate increased levels of CD3^+^ and CD8^+^ cells in the tumor stroma. Although there was a rising trend in the levels of CD3^+^ and CD8^+^ cells within the tumor in patients with postoperative PD, the observed difference had not achieved statistical significance. IFN-γ, mainly secreted by inflammatory cells including T cells, natural killer (NK) cells, and macrophages, significantly increases in postoperative PD patients. This rise further validates the heightened levels of inflammatory cells, CD3^+^ and CD8^+^ cells in patients with postoperative PD from an alternative standpoint. The findings suggest that TIC, SIC, CD3, CD8, and IFN-γ, may serve as novel prognostic indicators for assessing postoperative SD and PD in SCLC. The patients exhibiting an increased infiltration of inflammatory cells within the tumor paradoxically demonstrate a tendency to undergo postoperative PD, which may seem contradictory; however, relevant studies provide a foundation for understanding this phenomenon. SCLC tumor cells manifest an elevated mutational burden, suggesting their potential to elicit a robust T-cell response [4]. Nonetheless, it is noteworthy that SCLC ranks among the most extensively mutated cancer types, a factor that may significantly impact T cell responses [35, 43].

Lang et al. conducted an investigation on SCLC patients who underwent surgical resection and found that approximately 10% of tumor cells exhibited positive PD-L1 expression. Moreover, positive PD-L1 expression was evident in the tumor stroma in roughly 60% of cases, contributing significantly to a favorable impact on patient prognosis. Importantly, the authors did not discern a noteworthy correlation between PD-L1 expression and molecular subtypes of SCLC [44]. Yu et al. investigated the correlation between PD-L1 expression in SCLC and its anatomical locations (central and peripheral tumor regions) as well as the expression of TTF-1 (positive and negative expression). The study revealed a higher prevalence of PD-L1 expression in centrally located SCLC cases exhibiting positive TTF-1 expression. The findings suggest that PD-L1 expression serves as an unfavorable prognostic factor in SCLC, particularly when associated with vascular and lymphatic infiltration [45]. In this investigation, the majority of specimens derived from 18 cases of SCLC demonstrated a negative expression of PD-L1 in tumor cells. Conversely, in approximately 50% of the cases, there was a positive expression of PD-L1 in the tumor-infiltrating lymphocytes. This phenomenon may impede the cytotoxic capabilities of these lymphocytes against tumor cells, consequently playing a role in facilitating one of the mechanisms fostering immune escape in SCLC.

Tumor growth and metastasis hinge upon neovascularization, where microvessel density (MVD) in tumor tissues serves not only as a quantitative indicator of tumor vascular growth but also as a predictor for the trends in tumor growth, metastasis, and recurrence [46]. CD31, also known as PECAM-1, is a widely used marker for assessing MVD in tumor tissues. CD31 is implicated in mediating the adhesion of tumor cells to endothelial cells. In cases where tumor cells adhere to platelets, CD31 promotes their connection to endothelial cells, facilitating the interaction and stabilizing adhesion between tumor cells and endothelial cells. CD31 serves as a marker for vascular endothelial cells, and its expression level is indicative of neovascularization in tumor tissues. MVD denotes the counts of microvessels per unit area. Utilizing IHC staining of CD31 enables the quantitative determination of microvessel density in tumor tissues, facilitating the study of angiogenesis and tumor blood supply. Consequently, a significant association exists between CD31 and MVD, and the expression levels of CD31 serves as an indicator for evaluating the extent of angiogenesis in tumors [47]. This study used immunohistochemical methods to evaluate microvessel marker expression, specifically CD31, in 18 cases of SCLC tumors and the tumor stroma. The aim was to analyze the correlation between MVD and postoperative prognosis in SCLC patients. CD31 served as a microvessel marker in SCLC tissues, revealing varied CD31 expression levels in tumor specimens from SCLC surgeries. While patients with elevated CD31 expression showed an increasing trend in postoperative prognosis, unfortunately, the survival analysis outcomes lacked statistical significance. This may be due to the widespread upregulation of CD31 in SCLC patients, with few cases demonstrating low expression.

Our study has several limitations. The presence of selection bias, information bias, and institutional bias was inevitable, given the retrospective nature of this single-center study. Additionally, when employing retrospective questioning, the potential for recall bias exists, even though patients or their families may not have reported any memory difficulties. Owing to the lack of preoperative biopsy for pulmonary lesions or a preoperative pathological diagnosis confirming NSCLC, a significant portion of patients within the T3/4 SCLC cohort unexpectedly underwent surgical intervention. The presence of a limited number of patients in specific strata may pose challenges when attempting to perform rigorous statistical comparisons. Studies with a larger cohort, particularly focusing on T3/4 patients, could potentially mitigate bias, enhance statistical power, and validate the pertinent conclusions. We conducted IHC staining solely on samples obtained from 18 patients who underwent surgical resection, as limited by factors such as the availability of FFPE specimens, study duration, and research funding. Subsequent studies employing larger sample sizes are necessary to validate the significance of these prognostic indicators for surgically resected SCLC.

## Conclusion

In conclusion, the present study revealed that the patients with pT1-2N1M0 staging were a potential subgroup of SCLC patients who may benefit from surgery. Complete postoperative adjuvant therapy remains an independent factor promoting a better prognosis for SCLC patients undergoing surgical resection. Hence, conducting a thorough preoperative assessment of lymph node metastasis and administering complete postoperative adjuvant therapy are essential steps. These measures play a pivotal role in selecting suitable surgical candidates and improving prognosis. Moreover, CD3, CD8, IFN-γ, TIC, and SIC may serve as potential indicators for predicting the prognosis of surgically resected SCLC. Additionally, large, well-designed, prospective randomized trials are needed to further confirm these conclusions.

## Supplementary Information

Below is the link to the electronic supplementary material.

Supplementary file1 (XLSX 25 KB)

Supplementary file2 (XLSX 27 KB)

Supplementary file3 (XLSX 24 KB)

## Data Availability

The original contributions presented in the study are included in the article/Supplementary Material. Further inquiries can be directed to the corresponding author.

## Abbreviations

SCLC: Small cell lung cancer
IHC: Immunohistochemistry
SD: Stable disease
TIC: Tumor inflammatory cells
PD: Progressive disease
SIC: Stromal inflammatory cells
IARC: International Agency for Research on Cancer
RCTs: Randomized controlled trials
CT: Computed tomography
MDTs: Multidisciplinary teams
USTC: University of Science and Technology of China
IFN-γ: Interferon-gamma
TNF-α: Tumor necrosis factor-alpha
PD-L1: Programmed death ligand-1
OS: Overall survival
PFS: Progression-free survival
PET-CT: Positron Emission Tomography-Computed Tomography
EBUS-TBNA: Endobronchial ultrasound-guided transbronchial needle aspiration
NSCLC: Non-small cell lung cancer
PCI: Prophylactic cranial irradiation
WHO: World Health Organization
pT: Pathological T staging
pN: Pathological N staging
FFPE: Formalin-fixed paraffin-embedded
CTLs: Cytotoxic T lymphocytes
ROC: Receiver operating characteristic
AUC: Area under the curve
AJCC: American Joint Committee on Cancer
VALSG: Veterans Administration Lung Study Group
LS-SCLC: Limited-stage SCLC
ES-SCLC: Extensive-stage SCLC
ASCO: American Society of Clinical Oncology
NCCN: National Comprehensive Cancer Network
ESMO: European Society for Medical Oncology
EUS-FNA: Endoscopic ultrasound-guided fine needle aspiration
NCDB: National cancer database
NK: Natural killer
MVD: Microvessel density
